# Coincident polio and Ebola crises expose similar fault lines in the current global health regime

**DOI:** 10.1186/s13031-015-0058-1

**Published:** 2015-09-16

**Authors:** Philippe Calain, Caroline Abu Sa’Da

**Affiliations:** Research Unit on Humanitarian Stakes and Practices (UREPH), Médecins Sans Frontières, Rue de Lausanne 78, Geneva, 1211 Switzerland

**Keywords:** Global health, World Health Organization, Polio eradication, Ebola, Security, Militarization, Epidemic response, Humanitarian action, Disasters, International Health Regulations

## Abstract

**Background:**

In 2014, the World Health Organization (WHO) declared two "public health emergencies of international concern", in response to the worldwide polio situation and the Ebola epidemic in West Africa respectively. Both emergencies can be seen as testing moments, challenging the current model of epidemic governance, where two worldviews co-exist: global health security and humanitarian biomedicine.

**Discussion:**

The resurgence of polio and the spread of Ebola in 2014 have not only exposed the weaknesses of national health systems, but also the shortcomings of the current global health regime in dealing with transnational epidemic threats. These shortcomings are of three sorts. Firstly, the global health regime is fragmented and dominated by the domestic security priorities of industrialised nations. Secondly, the WHO has been constrained by constitutional country allegiances, crippling reforms and the limited impact of the (2005) International Health Regulations (IHR) framework. Thirdly, the securitization of infectious diseases and the militarization of humanitarian aid undermine the establishment of credible public health surveillance networks and the capacity to control epidemic threats.

**Summary:**

The securitization of communicable diseases has so far led foreign aid policies to sideline health systems. It has also been the source of ongoing misperceptions over the aims of global health initiatives. With its strict allegiance to Member States, the WHO mandate is problematic, particularly when it comes to controlling epidemic diseases. In this context, humanitarian medical organizations are expected to palliate the absence of public health services in the most destitute areas, particularly in conflict zones. The militarization of humanitarian aid itself threatens this fragile and imperfect equilibrium. None of the reforms announced by the WHO in the wake of the 68^th^ World Health Assembly address these fundamental issues.

**Electronic supplementary material:**

The online version of this article (doi:10.1186/s13031-015-0058-1) contains supplementary material, which is available to authorized users.

## Background

Drawing from international relations theory, the concepts of international regime, hegemonic stability and collective security have been used by scholars to analyze trends in the governance of global health. Focusing on epidemic diseases, Hoffman [1] defines the "global health security regime" as "the implicit or explicit principles, norms, rules and decision-making procedures by which international actors (including both states and civil society organizations) aim to protect their constituencies from the transmission of diseases from one area to another". Rather than a mere description of the roles and responsibilities of global health actors, this approach provides a better understanding of the complexity of epidemic governance. Furthermore, Lakoff [2] opposes as distinct regimes "global health security" and "humanitarian biomedicine" for their different visions of global health priorities. In our view, both global health security and humanitarian biomedicine inevitably co-exist in a complex political landscape defining the current "global health regime". Significantly, this regime has been put to the test in two coincident epidemic events of international dimensions, caused respectively by poliovirus and ebolavirus. Twice in 2014 (on May 5^th^ and August 8^th^ respectively) Dr. Margaret Chan, Director General of the World Health Organization (WHO), declared a public health emergency of international concern after consultation with an Emergency Committee of experts convened under the provisions of the revised (2005) International Health Regulations (IHR) [3]. Made in response to increasing alarms over the international spread of wild-type poliovirus, the first declaration was the latest sign that the success of polio eradication was still uncertain. The second announcement was a belated recognition that the Ebola epidemic in West Africa was unprecedented in magnitude and international spread.

According to Hoffman [1], the current global health regime has so far been characterised by expectations of international cooperation under the "hegemony" of the World Health Organization. It is being challenged by the limitations of the revised IHR (2005), the proliferation of global health security organizations, new instruments of foreign policy and new threats to health security. The IHR (2005) stand out as innovative among other health treaties. They oblige State Parties to notify defined public health threats and to limit unnecessary public health measures [4]. Yet, the temporary or standing recommendations issued by the WHO are not binding for State Parties, making the IHR (2005) weak instruments for outbreak response.

The mistrust of populations when public health actions are disconnected from local perspectives has been seen in recent circumstances, for example, the ongoing resistance to polio vaccination in Pakistan and the hostility of some communities towards measures to control Ebola in West Africa. Elsewhere, coincident political violence and civil wars are operational obstacles to polio eradication and other public health initiatives, which appear in a new geopolitical context, where public health is no longer seen as politically neutral. This is the case, for example, of ongoing conflicts in the Middle East. The current global health regime is poorly fit to meet such challenges, for at least three reasons, which we will further examine: (i) the encroachment of security policies on communicable diseases in general, and public health surveillance in particular, (ii) the constraining mandates of United Nations (UN) institutions, and (iii) the interference of security agendas with humanitarian action.

## Discussion

### Smallpox and polio eradication: different political epochs

The Global Polio Eradication Initiative launched in 1988 has been a remarkable endeavour prompted by the precedent of smallpox eradication. A public-private partnership endowed with considerable funding from private philanthropy, the initiative followed expected tracks until the early 2000s. This initial success led to the elimination of serotype 2 poliovirus in 1999, to the possible elimination of serotype 3 since November 2012 [5], and to wild-type serotype 1 remaining endemic in only three countries (Pakistan, Afghanistan and Nigeria) since 2012. Unfortunately, vaccination campaigns continue to be rejected by some communities and their traditional leaders in specific regions of overt or latent unrest. In northern Nigeria for example, religious leaders and authorities concerned about the safety of vaccines [6] and by the precedent of the Trovan drug trial [7] boycotted polio immunization in 2003–2004, resulting in the spread of polio to 20 countries [8]. In 2013, wild poliovirus spread from Nigeria to Cameroon and Somalia, while strains from Pakistan reached Iraq, Syria, Israel and Afghanistan [9]. In 2015, the propagation of polio seems to be halted in Iraq and Syria, but the situation in Pakistan and Afghanistan remains a major public health concern [10].

Non-binding temporary recommendations issued under the IHR (2005) are unlikely to solve the fundamental problems of "the last mile" of the Global Polio Eradication Initiative, which are not essentially technical, programmatic or even financial. More fundamentally, threats to the success of the Initiative are rooted in socio-cultural and political issues undermining confidence in vaccination programs [11]. When considering the final stages of the smallpox eradication campaign, it is barely surprising that social and cultural clashes could compromise the completion of a worldwide eradication campaign. In the 1970s, resistance to vaccination teams in India and Bangladesh was witnessed by Euro-American epidemiologists, who ultimately resorted to coercing villagers and intimidating local health care staff to achieve universal coverage [12]. What is new nowadays is the fact that polio remains endemic in zones of civil conflicts, where health care services are seen as symbols of foreign agendas [13]. Ongoing political violence exposes teams of polio vaccinators to being deliberately targeted by local insurgents, notably in northwestern Pakistan [14]. Accordingly, WHO country plans for Pakistan, Afghanistan and Nigeria have been adjusted to include more comprehensive public health strategies, security components and new communication tactics [15]. Ultimately, misinformation by obscurantist leaders or intimidation by extremist militants are only partial explanations for the local rejection of polio vaccination campaigns [16]. A more fundamental problem is that the global health regime is currently defined by security policies, which compromise the credibility of important public health initiatives of international dimensions.

### Securitization of infectious diseases

As a result of a prevailing focus on domestic security among industrialized countries, and in particular since the events in the USA on 9/11/2001, there has been a significant impact on the way global public health initiatives have been conceived.

Security interests in health became prominent in the 1990s with the recognition that communicable diseases (HIV/AIDS in particular) have far-reaching impact on trade and foreign affairs, beyond the strict realm of public health. In 2000, the UN Security Council passed Resolution 1308, concerned with the impact of HIV/AIDS on peacekeeping operations in Africa [17]. Following new concerns over bioterrorism, epidemic preparedness and response also became “securitized”, i.e. framed in terms of domestic and international security [18]. When applied to public health, the word “security” therefore carries a fundamental ambiguity about the exact values at stake [19]. Health security can either be understood in terms of protection of health, protection of trade and economy or as a matter of non-proliferation of biological weapons and counter-terrorism. This ambiguity has far reaching consequences, especially in the case of global public health surveillance.

### Box 1: glossary

**Table:** 

Biological Weapons Convention – a multilateral disarmament treaty prohibiting the development, production or stockpiling of bacteriological and toxin weapons.
Biosurveillance – " …the process of active data-gathering with appropriate analysis and interpretation of biosphere data that might relate to disease activity and threats to human or animal health – whether infectious, toxic, metabolic, or otherwise, and regardless of intentional or natural origin – in order to achieve early warning of health threats, early detection of health events, and overall situational awareness of disease activity" [20]. In contrast to public health surveillance, one explicit purpose of biosurveillance is to contribute to domestic security and measures to counter terrorist threats [21].
Dual use – the use of programmes or technologies for both civilian and military purposes.
Event-based surveillance – "…the organised collection, monitoring, assessment and interpretation of mainly unstructured ad hoc information regarding health events or risks, which may represent an acute risk to human health" [22].
International Health Regulations (2005) – A binding set of international regulations that requires States Parties to establish a credible national surveillance and response capacity and to notify a potentially wide range of events to the WHO on the basis of defined criteria indicating that the event may constitute a public health emergency of international concern [23].
Public health surveillance – "The systematic ongoing collection, collation and analysis of data for public health purposes and the timely dissemination of public health information for assessment and public health response as necessary" [23].
Securitization – framing the theory and the practice of a discipline (e.g. public health) as a matter of national security.

Global public health surveillance is an essential activity promoted under the IHR (2005) agenda [23], as well as one of the pillars of the Global Polio Eradication Initiative. After 2001, the security community became increasingly associated with the development of global public health surveillance, recognizing that early outbreak detection could identify or mitigate both natural and deliberate epidemic threats. From the beginning of the revision process of the IHR, this "dual use" argument has been a source of controversy. Whether the scope of the IHR (2005) includes the investigation of man-made outbreaks and consequently non-proliferation issues, is still open to interpretation. In practice the securitization of global public health surveillance is now pervasive and can be illustrated in a number of programmes deployed under private, national, international or supra-national initiatives (Table 1 and Additional file 1). Far from protecting global public health from securitization, the WHO Secretariat – through its partnerships and policies – has implicitly added support to the view that public health surveillance is primarily an instrument of national security instead of a foundation for outbreak prevention and control [24]. For example, Article VII 39 in the final document of the 7^th^ review conference of the Biological Weapons Convention (BWC) makes it clear that the IHR (2005) are instrumental to building surveillance and detection capacities pertaining to the BWC [25]. In this example, where the WHO is expected to provide the technical capacity to investigate suspicious outbreaks, securitization is counterproductive to public health goals [26]. The blurring of lines between security, disarmament and public health surveillance also appears in new surveillance networks sponsored by non-proliferation lobbies, in dual use technologies or in multilateral alliances (Table 1). Securitization underpins a subtle change of vocabulary, from the IHR (2005) "public health surveillance" [23] to "biosurveillance" [20]. The latter terminology not only reflects the increasing reliance on informal “event-based surveillance” [22] systems for outbreak detection, but also a shift from public health to domestic security concerns [21], [27]. For example, in 2004, the US National Biosurveillance Integration System was assigned to the Department of Homeland Security [28], while in 2008 the Biosurveillance Coordination Unit was established under the US CDC’s Coordination Office for Terrorism Preparedness and Emergency Response (COTPER) [29]. The proliferation of national and global biosurveillance initiatives has created a new industry that brings together public health institutions, academia, private security companies and the intelligence community. Counter to the argument that the goals of public health and national security converge over matters of epidemic control, security agendas actually prevail over public health achievements in the new health security doctrine. The fake vaccination campaign organised by the US Central Intelligence Agency (CIA) to help track Osama bin Laden showed how domestic security priorities can compromise trust in public health initiatives. In May 2011, a Pakistani doctor hired by the CIA conducted a hepatitis B vaccination campaign and allegedly managed to collect DNA samples from vaccinated children to confirm the presence of the Bin Laden family in their compound in the town of Abbottabad. The plot was clearly condemned by prominent international experts as damaging the trust in the polio eradication campaign and compromising its final success [30, 31].

**Table 1:** Examples of securitization of global public health surveillance: typology

Governance level	Securitizing agent	Initiatives or projects	Examples^a^
Private	Private philanthropy	Sponsoring the establishment of global surveillance networks	1. Nuclear Threat Initiative
National	USA (Security agencies, academia)	Development of event-based surveillance technology	2. Project Argus
	USA (Senate)	Capacity building, conditional aid to developing countries	3. US Global Pathogen Surveillance Act (2007)
International	USA (President)	Political and technical alliance	4. Global Health Security Agenda
	Some industrialised countries	Political alliance	5. Global Health Security Initiative
	Regional surveillance networks	Coordination of regional surveillance networks	6. CORDS
Supra-national	WHO	Technical resources for event verification	7. Biological Weapons Convention
	WHO/UNODA	Technical support to the UN Secretary-General	8. Memorandum of understanding

^a^Numbered examples and additional references are summarised in Additional File 1

### The paradox of UN mandates in civil conflict zones

The case of Syria is illustrative of the limitations imposed by UN mandates on the capacity of the WHO to ensure adequate public health responses in areas of civil conflicts [32]. UN agencies and the WHO in particular have been perceived taking sides with the Assad regime and disregarding the health needs of part of the Syrian population [33]. Polio vaccination activities have been very much at stake in this controversy. The Syrian Republic had not seen a case of polio since 1999. While polio probably reappeared as early as May 2013 in Deir al-Zour Province [34], an outbreak of acute flaccid paralysis was only confirmed as polio by Syrian authorities in October 2013. A controversy arose when the Syrian Government and the WHO country office were accused of delaying the confirmation of cases in areas sympathetic to the opposition [35, 36]. The outbreak seems to have been curbed in 2015, although the reliability of surveillance data is still disputed [34, 37]. Members of the new Islamic State insurgency support polio vaccination efforts [38], but it is doubtful if UN agencies alone can gain operational access and trust from all parties in conflicts. UN mandates and governance are indeed poorly adjusted to the fact that civil conflicts and public health crises are nowadays inevitably intertwined. The WHO is constitutionally constrained by its allegiance to Member States and cannot officially recognize opposition parties as operational partners. This is a problem in terms of neutrality, independence, legitimacy and access in situations of civil war. In contrast, some international humanitarian organizations have formal or informal legitimacy to operate regardless of political fractures and state funding. They cannot substitute for UN agencies, but their unequivocal neutrality and impartiality are assets to secure universal access to conflict zones and to remain credible. This is why the co-optation of humanitarian aid as an instrument of domestic security puts both humanitarian action and global public health initiatives in jeopardy.

### Interference of security agendas with humanitarian action

The setbacks of the Global Polio Eradication Initiative in its final stage could have been anticipated precisely in those conflict zones where access and trust are paramount. However, new counter-terrorism and foreign policies of Western coalitions are enmeshing humanitarian action into international security agendas, which can discredit the neutrality of all humanitarian actors, for example when relief and health care are provided to secure the acceptance of counter-insurgency operations [39, 40]. The securitization of global public health and the militarization of humanitarian action reflect the dominance of post-9/11 doctrines in the global health regime, and both trends combine to compromise the success of the Global Polio Eradication Initiative. This political complexity is not acknowledged by the WHO. In her opening statement at the 67th World Health Assembly, Dr. Chan attributed the recent downturn of international polio control to: “*Armed conflict that flies in the face of international humanitarian law. Civil unrest. Migrant populations. Weak border controls. Poor routine immunization coverage. Bans on vaccination by militant groups. And the targeted killing of polio workers*” [41]. Ironically, just ahead of that opening session, the CIA implicitly acknowledged some responsibility by announcing that the agency was ending the use of vaccination programmes in its spying operations [42].

### Global health governance after the Ebola epidemic

A consensus has emerged to say that the disastrous situation in Guinea, Liberia and Sierra Leone reflects the disarray of national health systems and a vacuum in global health governance [43, 44]. With a much delayed and fragmented regional response, the case is an archetype of the global health security regime in several respects. What ultimately triggered the international mobilisation of adequate resources was the realisation that the epidemic could easily spread out of Africa and represented a common threat to international peace and security. In September 2014, three major political decisions followed this reasoning. Firstly, the UN Security Council adopted Resolution 2177, acknowledging that the Ebola outbreak in Western Africa constituted a threat to international peace and security. Secondly, the US administration deployed some 3,000 military personnel in Liberia to reinforce outbreak-control measures. Although such an initiative was generally acclaimed as a valuable contribution to controlling a catastrophic situation, some scholars see it as yet another example of the militarization of humanitarian aid [45, 46]. Thirdly, a UN Mission for Ebola Emergency Response (UNMEER) was established in Ghana. Its regional mandate and authority are limited, but as the first UN emergency health mission, UNMEER might become a precedent for a new transnational outbreak governance system, thus marginalising the IHR (2005) framework of non-binding recommendations.

It is uncertain how the current global health security regime will evolve [1], particularly after the epidemic crises of 2014–2015. Notwithstanding an evaluation of the performance of the IHR (2005), the ‘reforms’ proposed by the WHO [47, 48] in the wake of the 68^th^ World Health Assembly consist in: (i) integrating WHO outbreak and emergency response units, (ii) the creation of a global health-emergency workforce, (iii) setting up an emergency contingency fund, and (iv) advancing the research and development of medical products for infectious diseases of epidemic potential, and (v) strengthening health systems. One could muse over the fact that such measures are belated, obvious or simply represent an attempt to resurrect similar assets dismantled by a recent round of crippling reforms. More than with circumstantial resolutions, global health would be served by genuine reforms of the current regime, emphasizing universal health values instead of security and diplomacy interests. This would entail a new constitutional mandate for the WHO. Reflecting on the future of global health governance, Lawrence Gostin has called for a new Framework Convention on Global Health [49]. With a bold departure from the UN governance model, a new and more credible global health regime could be built upon such a convention, by transcending narrow State interests. The core of this new architecture would make the WHO akin to the International Committee of the Red Cross with its supranational mandate, with political independence and a better capacity to react to global health crises.

### Summary

The setbacks of the Global Polio Eradication Initiative and the delayed control of the Ebola epidemic in West Africa reflect a fragmented approach to outbreak preparedness. More broadly, they point to profound flaws in the current regime of global health governance, which is guided by foreign affairs and security policies. The securitization of communicable diseases has so far led foreign aid policies to sideline health systems. It has also been the source of ongoing misperceptions over the aims of global health initiatives. With its strict allegiance to Member States, the WHO mandate is problematic, particularly when it comes to controlling epidemic diseases. In this context, humanitarian medical organizations are expected to palliate the absence of public health services in the most destitute areas, particularly in conflict zones. The militarization of humanitarian aid itself threatens this fragile and imperfect equilibrium. None of the reforms announced by the WHO in the wake of the 68^th^ World Health Assembly address these fundamental issues.

## Additional file

Additional file 1:
**Examples of securitization of global public health surveillance: overview of selected initiatives or projects.** (DOC 50 kb)

## Abbreviations

BWC: Biological Weapons Convention
CIA: US Central Intelligence Agency
COTPER: Coordination Office for Terrorism Preparedness and Emergency Response
IHR: International Health Regulations
UN: United Nations
UNMEER: UN Mission for Ebola Emergency Response
USA: United States of America
WHO: World Health Organization
