# Supplementary material for: Coincident polio and Ebola crises expose similar fault lines in the current global health regime
Source: Confl Health. 2015 Sep 16;9:29. doi: 10.1186/s13031-015-0058-1 (PMC4572646; doi:10.1186/s13031-015-0058-1)
Supplement: Additional file 1: — Examples of securitization of global public health surveillance: overview of selected initiatives or projects. (DOC 50 kb) [file 13031_2015_58_MOESM1_ESM.doc]

**Table 1. Examples of securitization of global public health surveillance: typology**

| **Governance level** | **Securitizing agent** | **Initiatives or projects** | **Examples (*)** |
| --- | --- | --- | --- |
| Private | Private philanthropy | Sponsoring the establishment of global surveillance networks | 1. Nuclear Threat Initiative |
| National | USA (Security agencies, academia)  USA (Senate) | Development of event-based surveillance technology  Capacity building, conditional aid to developing countries | 1. Project Argus 2. US Global Pathogen Surveillance Act (2007) |
| International | USA (President)  Some industrialised countries  Regional surveillance networks | Political and technical alliance  Political alliance  Coordination of regional surveillance networks | 1. Global Health Security Agenda 2. Global Health Security Initiative 3. CORDS |
| Supra-national | WHO  WHO/UNODA | Technical resources for event verification  Technical support to the UN Secretary-General | 1. Biological Weapons Convention 2. Memorandum of understanding |

**(*) Numbered examples and additional references are summarised in Supplementary File 1.**

**Supplementary file 1. Examples of securitization of global public health surveillance: overview of selected initiatives or projects**

1. *Nuclear Threat Initiative (NTI)*

“The Nuclear Threat Initiative (NTI) is a nonprofit, nonpartisan organization with a mission to strengthen global security by reducing the risk of use and preventing the spread of nuclear, biological, and chemical weapons and to work to build the trust, transparency, and security that are preconditions to the ultimate fulfilment of the Non-Proliferation Treaty’s goals and ambitions.”

Nuclear Threat Initiative. Understanding biological threats; 2014. Available from: <http://www.nti.org/threats/biological/projects/>

1. *Project Argus Biosurveillance System*

Hosted by Georgetown University and funded by intelligence community agencies, project Argus is “…a prototype biosurveillance system designed to detect and track biological events that may threaten human, plant, and animal health globally. The approach is based on monitoring social disruption evident in local, native-language media reports around the world”. The outreach of project Argus for data gathering is global, but monitoring inside of the US territory is not legally permitted.

Building USG interagency collaboration through global health engagement. CDC Global Health E-brief; first quarter 2008.

Available from: <http://www.cdc.gov/washington/EGlobalHealthEditions/E-brief_first_quarter_2008.pdf>

US Senate Committee on Homeland Security and Governmental Affairs. Oversight of Government Management Subcommittee. Statement by James M. Wilson V, MD, Research Faculty of Department of Pediatrics and Director of Division of Integrated Biodefense, Imaging Science and Information Systems (ISIS) Center at Georgetown University Before the Senate Homeland Security & Government Affairs Subcommittee on Oversight of Government Management, the Federal Workforce, and the District of Columbia; October 4, 2007. Available from: <http://www.hsgac.senate.gov/subcommittees/oversight-of-government-management/hearings/forestalling-the-coming-pandemic-infectious-disease-surveillance-overseas>

1. *US Global Pathogen Surveillance Act*

A bill introduced to the US Senate to “help strengthen developing countries' abilities to identify and track pathogens that could be indicators of dangerous disease outbreaks-either naturally-occurring or deliberately released.” The bill failed to pass in 2007.

Mitka M. Global pathogen surveillance. *JAMA* 2006;295(6):617.

1. *Global Health Security Agenda*

Launched in 2014 by President Obama, the Global Health Security Agenda is “an effort between the U.S. government, other nations, international organizations and public and private stakeholders, to accelerate progress toward a world safe and secure from infectious disease threats and to promote global health security as an international security priority.” Its promoters “are coordinating closely with the World Health Organization, World Organization for Animal Health, and Food and Agriculture Organization of the United Nations to accelerate progress toward achieving the goals of the IHRs and facilitating other global health security measures.”

Global health security. United States Department of Health and Human Services; 2014. Available from: <http://www.globalhealth.gov/global-health-topics/global-health-security/index.html>

1. *Global Health Security Initiative (GHSI)*

“... an informal, international partnership among like-minded countries to strengthen health preparedness and response globally to threats of biological, chemical, radio-nuclear terrorism (CBRN) and pandemic influenza” established in 2001 by Canada, France, Germany, Italy, Japan, Mexico, the United Kingdom, the United States and the European Commission. The GHSI convenes annual meetings at ministerial level. The World Health Organization serves as an expert advisor to the GHSI.

Global Health Security Initiative; 2014. Available from: <http://www.ghsi.ca/english/index.asp>

1. *Connecting Organizations for Regional Disease Surveillance (CORDS)*

Initiated and partly funded by NTI, CORDS serves as a platform for a global disease surveillance network.  The network is designed to enhance communication and cooperation as regional disease networks, states and global health organizations respond to future pandemic outbreaks and biological weapon threats.

Gresham L, Smolinski M, Suphunchaimat R, Kimball AM, Wibulpolprasert S. Creating a global dialogue on infectious disease surveillance: Connecting Organizations for Regional Disease Surveillance (CORDS). *Emerging Health Threats* 2013;6,19912. Available from: <http://www.eht-journal.net/index.php/ehtj/article/view/19912>

1. *Biological Weapons Convention (BWC)*

*Biological Weapons Convention (BWC) –*The Convention on the Prohibition of the Development, Production and Stockpiling of Bacteriological (Biological) and Toxin Weapons and on their Destruction, commonly known as the Biological Weapons Convention (BWC) or Biological and Toxin Weapons Convention (BTWC) entered into force in 1975. It was the first multilateral disarmament treaty banning an entire category of weapons. It effectively prohibits the development, production, acquisition, transfer, retention, stockpiling and use of biological and toxin weapons and is a key element in the international community’s efforts to address the proliferation of weapons of mass destruction.

UNOG. The Biological Weapons Convention. United Nations Office at Geneva (2014). Available from: [http://www.unog.ch/80256EE600585943/%28httpPages%29/04FBBDD6315AC720C1257180004B1B2F?OpenDocument](http://www.unog.ch/80256EE600585943/(httpPages)/04FBBDD6315AC720C1257180004B1B2F?OpenDocument)

1. *United Nations Office for Disarmament Affairs (UNODA)*

In a Memorandum of Understanding with UNODA, the WHO Secretariat commits to “provide technical support in assessing the public health, clinical, and event-specific health aspects of an alleged use [of chemical, biological or toxin weapons] that are brought to the attention of the Secretary –General”.

United Nations Office for Disarmament Affairs. Secretary-General’s mechanism for investigation of alleged use of chemical and biological weapons; 2014. Available from: <http://www.un.org/disarmament/WMD/Secretary-General_Mechanism/UN_WHO_MOU_2011.pdf>
